# Dichotomous Temperature Response in the Electronic Structure of Epitaxially Grown Altermagnet MnTe

**DOI:** 10.1021/acs.nanolett.5c01158

**Published:** 2025-05-19

**Authors:** Ji-Eun Lee, Yong Zhong, Qile Li, Mark T. Edmonds, Zhi-Xun Shen, Choongyu Hwang, Sung-Kwan Mo

**Affiliations:** † Advanced Light Source, 1666 Lawrence Berkeley National Laboratory, Berkeley, California 94720, United States; ‡ Max Planck POSTECH Center for Complex Phase Materials, 34996 Pohang University of Science and Technology, Pohang 37673, Korea; § Stanford Institute for Materials and Energy Sciences, SLAC National Accelerator Laboratory, Menlo Park, California 94025, United States; ∥ Department of Applied Physics, 6429 Stanford University, Stanford, California 94305, United States; ⊥ School of Physics and Astronomy, 2541 Monash University, Clayton, Victoria 3168, Australia; # ANFF-VIC Technology Fellow, Melbourne Centre for Nanofabrication, Victorian Node of the Australian National Fabrication Facility, Clayton, Victoria 3168, Australia; ¶ Department of Physics, Stanford University, Stanford, California 94305, United States; ○ Department of Physics, Pusan National University, Busan 46241, Korea; △ Quantum Matter Core-Facility, Pusan National University, Busan 46241, Korea

**Keywords:** MnTe, altermagnet, electronic band structures, molecular beam epitaxy, ARPES

## Abstract

The altermagnet candidate MnTe has recently gained significant interest due to its unconventional magnetic ordering. One of the key features of altermagnetism is the momentum-dependent spin-split band and its temperature-dependent evolution. Yet a fully momentum-resolved experimental investigation, including out-of-plane direction, is still lacking. Here, we systematically investigate the electronic structure of epitaxially grown MnTe by using angle-resolved photoemission spectroscopy (ARPES). Our photon-energy-dependent ARPES data reveal significant out-of-plane dispersions consistent with previous theoretical calculations. More interestingly, we identify two distinct temperature-dependent electronic band structure evolutions at different out-of-plane momentum positions: momentum-dependent energy shifts at the nodal plane and substantial spectral weight suppression at the off-nodal plane. These findings may suggest the importance of considering both the itinerant and localized nature of the magnetic ordering and momentum-dependent interactions. Our work provides crucial insights into the complex correlation between momentum, temperature, and electronic structure in MnTe, contributing to a deeper understanding of altermagnetism.

## Graphical abstract

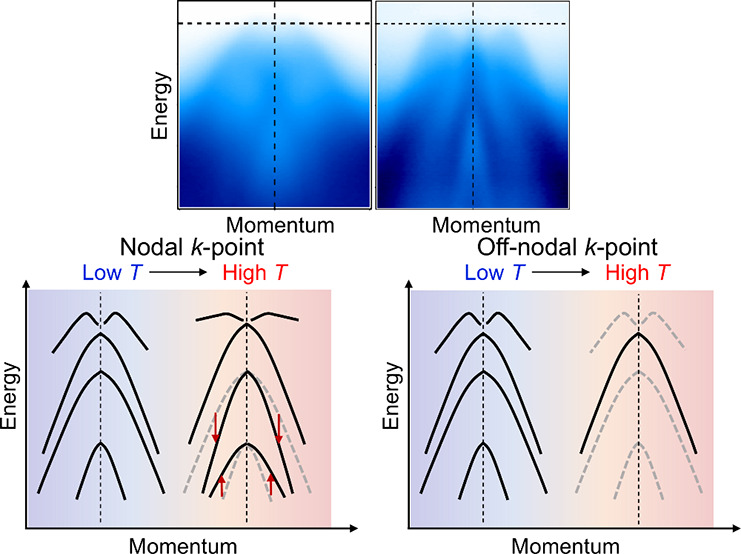

The magnetism in condensed matter is typically classified into various spin-ordered ground states, such as ferromagnetism, antiferromagnetism, ferrimagnetism, and noncollinear spin structures, based on the spin correlation and magnetization ordering.^1,2^ Recent theoretical progress has unveiled a new type of magnetism dubbed “altermagnetism”, which exhibits unconventional time-reversal symmetry (TRS) breaking, even in compensated collinear antiferromagnetic ordering with vanishing net magnetization.^3–5^ In contrast to conventional antiferromagnet (AFM), where opposite spin sublattices are linked by translation or inversion symmetries, the opposite spin densities are connected by a rotational symmetry in altermagnets, leading to the breaking of TRS. Consequently, altermagnets possess unconventional electronic structures characterized by anisotropic and momentum-dependent nonrelativistic spin splitting, even in the absence of spin–orbit coupling (SOC). The novel magnetic order and electronic structure lead to exotic spin phenomena such as unconventional anomalous Hall effect, circular dichroism (CD), and Kerr effect.^6–17^

One of the most intensively investigated altermagnet candidates is centrosymmetric α-MnTe. Due to the presence of the sixfold lattice rotational symmetry (*C*_6_) connecting two sublattices with opposite spins, it has been theoretically predicted to exhibit *g*-wave type altermagnetism and unconventional spin-polarization, whose signatures are recently observed in angle-resolved photoemission spectroscopy (ARPES), anomalous Hall effect, and X-ray magnetic circular dichroism (XMCD) measurements.^3,4,7,9,12,14,15^ Particularly, ARPES studies have been considered to provide critical evidence of altermagnetism by directly visualizing the temperature-dependent band splitting and spin polarization.^12,14,15^ However, existing experimental results are mostly limited to single or a few selected *k*_*z*_ values, lacking systematic three-dimensional (3D) mapping of the electronic structure. Furthermore, none have integrated both *k*_*z*_ evolution and temperature dependence into a comprehensive analysis, leaving the interplay between the temperature- and momentum-dependent electronic structure unaddressed. Such momentum resolution is crucial to discern the distinct nature of altermagnetism at different momentum planes along *k*_*z*_: relativistic spin-splitting induced by SOC at nodal planes (weak altermagnetism) vs large alternating magnetic spin-splitting at off-nodal planes (strong altermagnetism).^12^ Moreover, distinct temperature-dependent electronic structure evolution is expected theoretically^14,18^ depending on the nature of the altermagnetism.

In this study, we report the electronic structure of epitaxially grown MnTe on Si(111), with a particular focus on *k*_*z*_-dependent electronic structure and temperature-dependent evolution of electronic structure at different *k*_*z*_ momenta. The synchrotron-based ARPES data with tunable photon energy provide a full 3D view of the electronic structure of epitaxially grown MnTe, in which we found a significant *k*_*z*_-dependence. We also notice two distinct temperature-dependent band structure evolutions at different out-of-plane momenta. In the nodal plane, we observe temperature- and momentum-dependent energy shifts of individual bands. On the contrary, the off-nodal plane exhibits minor changes in the band dispersions themselves but a pronounced suppression of spectral weight near the Fermi energy (*E*_F_) with increasing temperature. The temperature-induced spectral weight reduction is beyond the expected itinerant picture of altermagnetism, which may suggest the important role of local magnetic moments in the altermagnetism and electronic structure of MnTe. Our work presents temperature-dependent analysis across multiple *k*_*z*_ values, offering new insights beyond previous studies. These findings provide a comprehensive exploration of the electronic structure of the altermagnet candidate MnTe thin film, enhancing the comprehension of its microscopic origin and unlocking the opportunity for advanced nanoelectronic applications.

MnTe crystallizes in the α-phase of a NiAs-type hexagonal structure with the space group P6_3_/*mmc*, where two magnetic sublattices are connected by a sixfold screw axis along the [0001] *c*-axis. Mn atoms are ferromagnetically ordered along the in-plane direction while stacked antiferromagnetically along the out-of-plane direction. This collinear-compensated magnetic order is characterized by two rotational symmetries: sixfold rotation (C_6*z*_t_1/2_) in real space and twofold rotation (C_2*y*_) in spin space, connecting the two inequivalent spin sublattices denoted as Mn A site and Mn B site (Figure 1a). Despite the absence of net magnetization, MnTe exhibits broken time-reversal symmetry resulting from nonrelativistic spin-group symmetry.^3,4^ Consequently, in the absence of SOC, the electronic structure exhibits large spin-splitting across the momentum space. However, there are four specific planes where spin degeneracy is preserved: one at the *k*_*z*_ = 0 plane and three along the Γ-K directions. These nodal planes remain spin-degenerate without SOC.^12,14^ In this regard, the Γ-M direction offers a favorable geometry for investigating the evolution of altermagnetic spin splitting as a function of out-of-momentum (*k*_*z*_), since Kramers degeneracy is lifted even in the absence of SOC. The bulk and its projected surface Brillouin zones (BZs) of MnTe are presented in Figure 1b.

**Figure 1:**
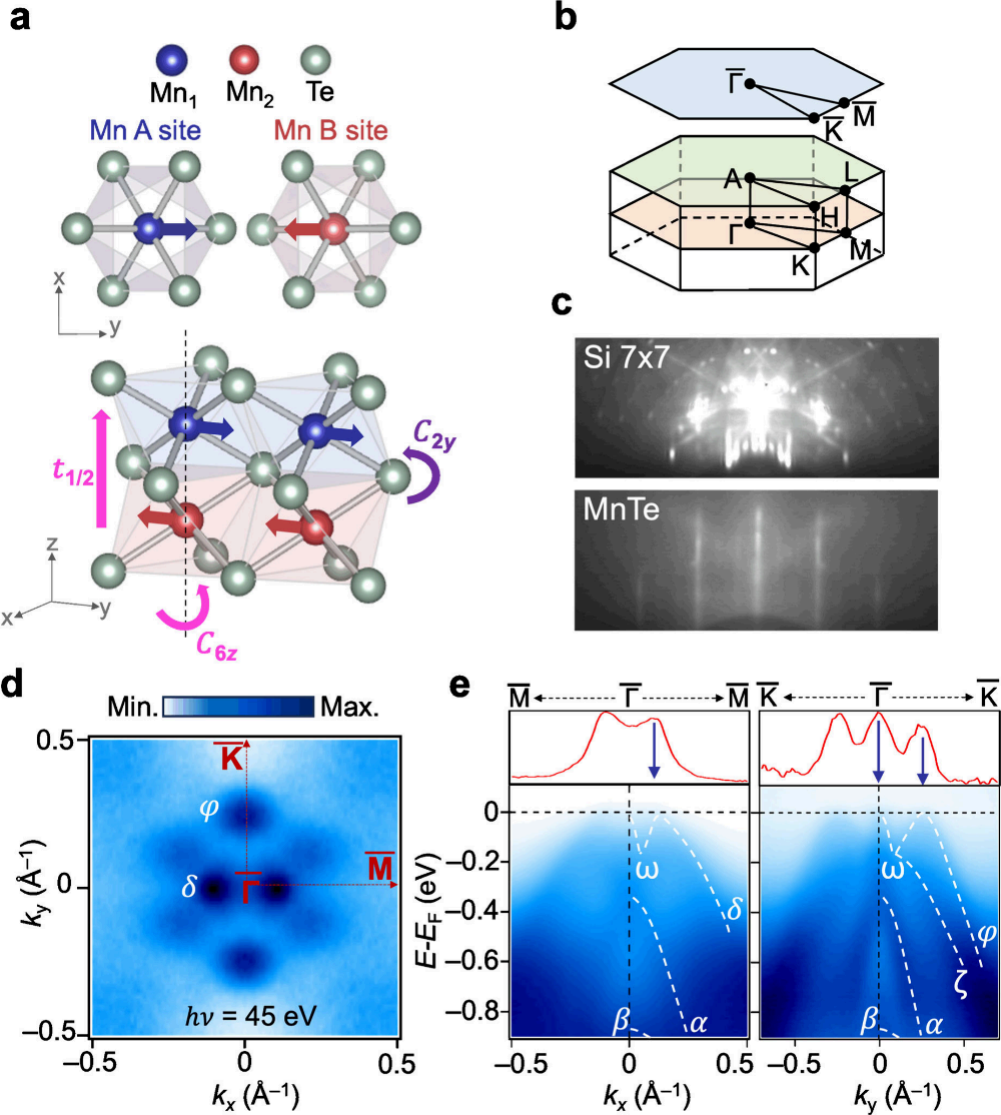
Characteristics of epitaxially grown MnTe. (a) Crystal structure of MnTe with two inequivalent spin configurations of Mn spin-sublattices (Mn A and Mn B sites). Its two spin-sublattices are connected by sixfold rotation C_6*z*_t_1/2_ combined with C_2*y*_ spin rotation. (b) Three-dimensional (3D) and corresponding projected two-dimensional (2D) Brillouin zones with high-symmetry points labeled. (c) *In-situ* RHEED patterns of a Si (111) 7 × 7 substrate and a MnTe thin film grown on the substrate. (d) ARPES measurement of FS using a photon energy of 45 eV at 20 K with linear vertical (LV) polarization. (e) ARPES intensity cuts along the Γ̅-M̅ and Γ̅-K̅ directions using a photon energy of 45 eV at 20 K with LV polarization. The top panels show MDCs at *E* – *E*_F_ = 0 eV, highlighting the band features near *E*_F_. The white-dashed curves describe the characteristic bands (δ, φ, α, β, ω, and ζ) of MnTe.

MnTe thin films with a thickness of 8 monolayers (MLs) have been successfully grown on a Si(111) substrate with 7 × 7 superstructure using molecular beam epitaxy (MBE). Figure 1c shows the reflection high-energy electron diffraction (RHEED) patterns of the Si 7 × 7 substrate and high-quality single-crystal MnTe, respectively. We also observe the Te 4*d* and Mn 3*p* characteristic peaks in the core-level spectrum (Figure S1).

Figure 1d and e represent the overall electronic structures of MnTe. The prominent features of the Fermi surface (FS) measured with 45 eV photon energy are two distinct sets of dots derived from the two hole bands: the φ band along the Γ̅-K̅ direction and the δ band along the Γ̅-M̅ direction. The twofold symmetry of the δ band is more clearly pronounced in polarization-dependent ARPES data (Figure S2), by tracking the dumbbell-like constant energy contour at higher binding energy. The intensity of the FS component derived from the ω band right at the Γ̅ point is heavily suppressed in the particular geometry in which the FS is measured. Increasing the out-of-plane component of the incident light polarization suppresses other features of the FS and makes the dot-like FS piece at the center of the BZ more clearly visible (Figure S3). Our measured FS and high symmetry cuts exhibit natural hole-doping in MnTe/Si(111), consistent with the *p*-type semiconductor characteristics found in previous studies.^12,14,15,19^ The most pronounced bands in ARPES measurements along high-symmetry directions are traced with white dotted curves (δ, φ, α, β, ω, and ζ) in Figure 1e. The bands along the Γ̅-M̅ and Γ̅-K̅ direction bear close similarity to those reported in previous ARPES studies^12,18,20^ on thick films and first-principles calculations^18,21^ of the bulk, suggesting that the electronic structure of an 8 ML sample is a representative of bulk similar to other transition metal dichalcogenides thin films.^22^ The δ and φ bands exhibit hump-like features with high spectral intensity near *E*_F_, as clearly identified in the momentum distribution curves (MDCs). The ω band is more clearly observable in the Γ̅-K̅ cut, while its relative intensity is heavily sensitive to the incident light polarization (Figure S3) and becomes more prominent only at specific *k*_*z*_ values, consistent with the previous ARPES results reporting strong *k*_*z*_-dependence.^15^ A similar hole-like band near Fermi energy (*E*_F_) centered at the Γ̅-point has been observed in the ARPES studies on bulk^15^ and thin film^20^ of MnTe. While the α band shows only a minor difference in its intensity and the effective mass along two different high-symmetry directions, the ζ band displays a significant intensity difference. It is only clearly visible along the Γ̅-K̅ direction, consistent with previous results^20^ measured on MnTe grown on an InP substrate. As a result, the spin-splitting near *E*_F_ is clearly visible only along the Γ̅-K̅ direction.^20^ The spin-splitting is difficult to resolve along Γ̅-M̅, in contrast to some of the prior results,^12^ due to the suppression of photoemission signal in our particular photon energy, polarization, and measurement geometry. The strong polarization dependence of δ and ω bands, against both in-plane azimuthal orientation and out-of-plane orientation of the linear polarization vector of the incident light with respect to the sample high-symmetry planes, hints at the orbital characters of these bands.^23,24^ Furthermore, first-principles calculations reveal that the valence band arises from the hybridization of Mn 3*d* and Te 5*p* orbitals, further supporting the interpretation that orbital interactions play a key role in the low-energy electronic structure.^21,25^

To acquire a complete picture of the momentum-resolved electronic band structure of MnTe, it is essential to conduct photon energy-dependent ARPES measurements to probe the out-of-plane momentum. Figure 2 summarizes photon energy-dependent ARPES data along the projected Γ̅-M̅ direction, corresponding to *k*_*z*_ values ranging from 0 to 0.5 Å^–1^. We observed a clear *k*_*z*_ dispersion with the experimentally determined inner potential of 10 eV (Figure S4). We note that the inner potential reported in various ARPES studies are rather scattered.^14,15,18^ This may be due to the differences in experimental conditions such as photon energy, measurement geometry, and surface quality, which bar a consistent determination of *k*_*z*_ assignment across different studies. At *k*_*z*_ = 0 Å^–1^, four characteristic bands are identified: the δ band near *E*_F_ and three parabolic hole bands labeled as α, β, and γ at higher binding energies. These bands are qualitatively consistent with previous theoretical calculations for MnTe.^14,18,21,26^ We observe that the δ band, which exhibits a hump structure at *k*_*z*_ = 0 Å^–1^, gradually shifts toward the higher binding energy with decreasing spectral intensity as the *k*_*z*_ value increases (Figure 2a and b). Simultaneously, the α band shifts toward lower binding energies, while the β and γ bands shift toward higher binding energies with increasing *k*_*z*_ (Figure 2c). This evolution alters the position of the valence band maximum (VBM) along the Γ̅-M̅ direction, shifting from *k*_*x*_ = 0.16 Å^–1^ at *k*_*z*_ = 0 Å^–1^ to *k*_*x*_ = 0 Å^–1^ at *k*_*z*_ = 0.5 Å^–1^. Our ARPES measurements clearly visualize a strong *k*_*z*_-dependence in the electronic structure of MnTe, consistent with previous theoretical calculations.^26^ We also observe noticeable changes in the ARPES spectral intensity, depending on the photon energy. For example, the α and γ bands can be identified across all the *k*_*z*_ values, whereas the intensity of the β band varies significantly with photon energies (Figure 2a and c).

**Figure 2:**
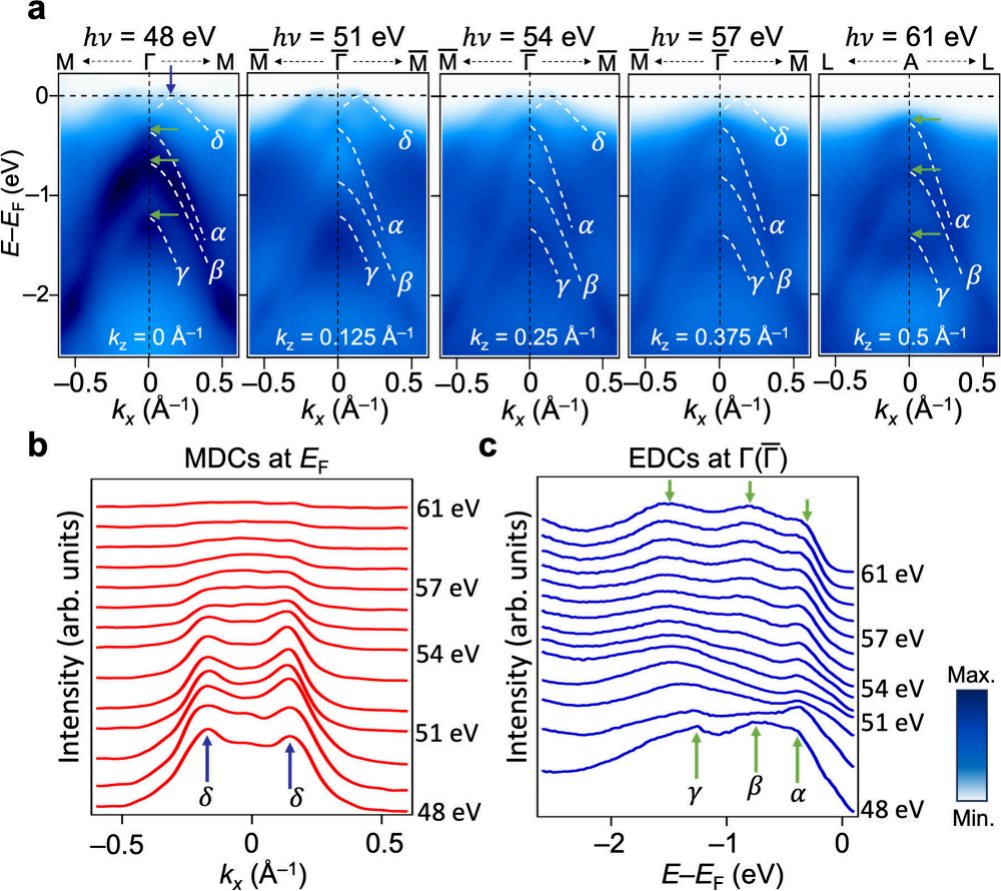
Photon energy dependence of ARPES spectra at 20 K. (a) ARPES intensity cuts along the Γ̅-M̅ direction with *k*_*z*_ = 0, 0.125, 0.25, 0.375, and 0.5 Å^–1^, respectively. The white dotted curves are guides to eyes for the characteristic bands (δ, α, β, and γ) of MnTe. (b) Photon energy-dependent MDCs taken at *E* – *E*_F_ = 0 eV. The blue arrows indicate the intensities from the δ band. (c) Photon energy-dependent energy distribution curves (EDCs) taken at the Γ­(Γ̅) point. The green arrows indicate the α, β, and γ bands.

Now that a better understanding of the *k*_*z*_-dependent electronic structure has been established, two representative *k*_*z*_ positions, *k*_*z*_ = 0 Å^–1^ (nodal plane) and 0.28 Å^–1^ (off-nodal plane), are chosen to investigate the temperature-dependent electronic structure variation. To ensure the reliability of the results, the experiments were repeatedly performed for multiple samples and temperature cycling processes, which yielded reproducible results. The ARPES results measured at temperatures ranging from 70 to 250 K are shown in Figure 3. In the lower panels, the second-derivative ARPES intensity maps are plotted to improve the visibility of low-intensity band features.

**Figure 3:**
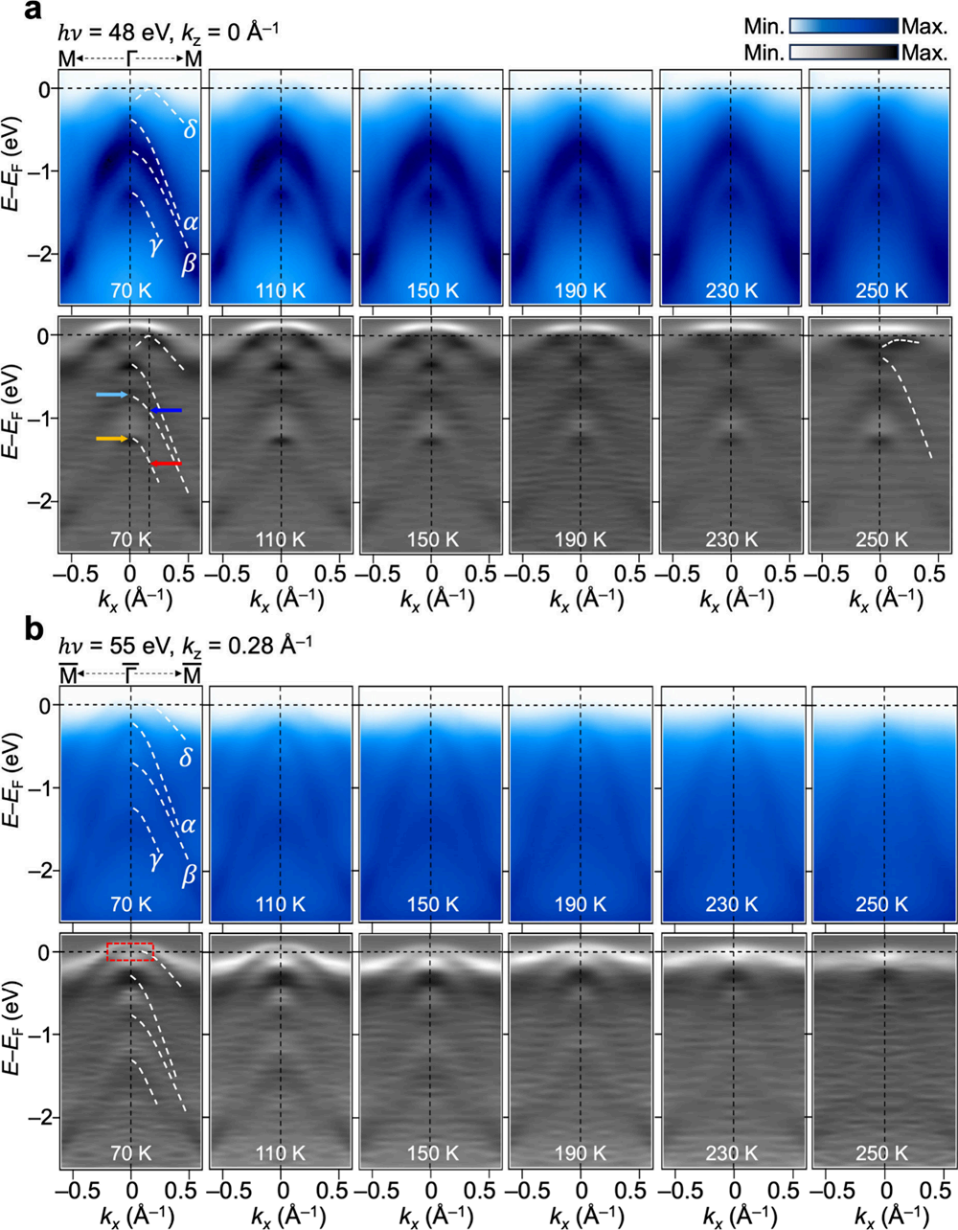
Temperature dependence of ARPES spectra at different *k*_*z*_ values. (a) Temperature dependence of ARPES intensity cuts along the Γ-M directions with *k*_*z*_ = 0 Å^–1^ at temperatures of 70, 110, 150, 190, 230, and 250 K. The white-dashed curves overlaid on the spectra represent characteristics band features of MnTe, as previously illustrated in Figure 2. Two black-dashed lines are located at *k*_*x*_ = 0 Å^–1^ and 0.16 Å^–1^, highlighting the β and γ bands at each *k*_*x*_ point, as indicated by yellow, cyan, red, and blue arrows. The lower panels provide the second derivatives of the bands, offering enhanced visibility of band features. (b) Temperature dependence of ARPES intensity cuts along the Γ̅-M̅ directions at *k*_*z*_ = 0.28 Å^–1^, along with the second derivatives. The red-dashed rectangle indicates the momentum-energy integrated area, which will be further discussed in Figure 4b.

At the nodal plane (*k*_*z*_ = 0 Å^–1^), the δ, β, and γ bands show a temperature-dependent evolution. As shown in Figure 3a, the curvature of the δ band changes with temperature, becoming flatter with an increasing temperature. The top of the α band at the Γ point shifts toward lower binding energies with increasing temperature (Figure S5). This observation is consistent with the ARPES and theoretical study^18^ made close to the nodal *k*_*z*_ position. Since the top of the α band remains spin-degenerate across the magnetic transition, the observed shift is unlikely to be of magnetic origin. At the off-nodal planes (*k*_*z*_ = 0.28, −0.09 Å^–1^), the most pronounced feature of the temperature-dependent ARPES data is a substantial decrease in spectral weight near *E*_F_ with increasing temperature, as presented in Figure 3b and Figure S6. One identifies two peaks in the MDCs at *E*_F_ originating from the δ band at low temperatures (Figure S7c), which gradually decrease in intensity to make a gapped state as the temperature rises to 250 K.

These observations highlight significant differences in temperature responses between the nodal and off-nodal planes: while the spectral intensity of the δ band at the nodal plane is nearly unchanged with increasing temperature, it shows a notable suppression, indicating a potential transition into a gapped state at the off-nodal plane. Additionally, at the off-nodal plane, the intensity of the β and γ bands become weaker with increasing temperature while the intensity of the α band remains strong, raising questions about whether this is due to differences in orbital character, band hybridization, or coupling strength to local spin fluctuations. In contrast, these bands exhibit distinctive energy shifts in the nodal plane.

To better visualize and quantify the temperature dependence of the electronic structures, a more detailed analysis is shown in Figure 4. We have extracted EDCs at *k*_*x*_ = 0 Å^–1^ and *k*_*x*_ = 0.16 Å^–1^, two dashed lines overlaid on second-derivative plots in Figure 3a, and followed their temperature evolutions (Figure S7a and b). Two distinct peaks (the β and γ band) are observed, and their temperature dependence differs depending on the parallel momenta (*k*_*x*_ positions). At *k*_*x*_ = 0 Å^–1^, the bands exhibit virtually no change with temperature, as highlighted by the yellow and cyan arrows (Figure S7a). In contrast, away from the center of the Brillouin zone (*k*_*x*_ = 0.16 Å^–1^), these bands shift with the temperature (Figure S7b). Specifically, the β band is shifted by ∼29 meV toward higher binding energies, and the γ band is shifted by ∼75 meV toward lower binding energies (Figure 4a and Figure S7a-b). To quantify these energy shifts, we performed multipeak fitting using both a Lorentzian function and a linear function as background models (Figures S8 and S9). Figure 4a presents the results using the Lorentzian background model. The consistency between these two approaches confirms the reliability and robustness of our analysis.

**Figure 4:**
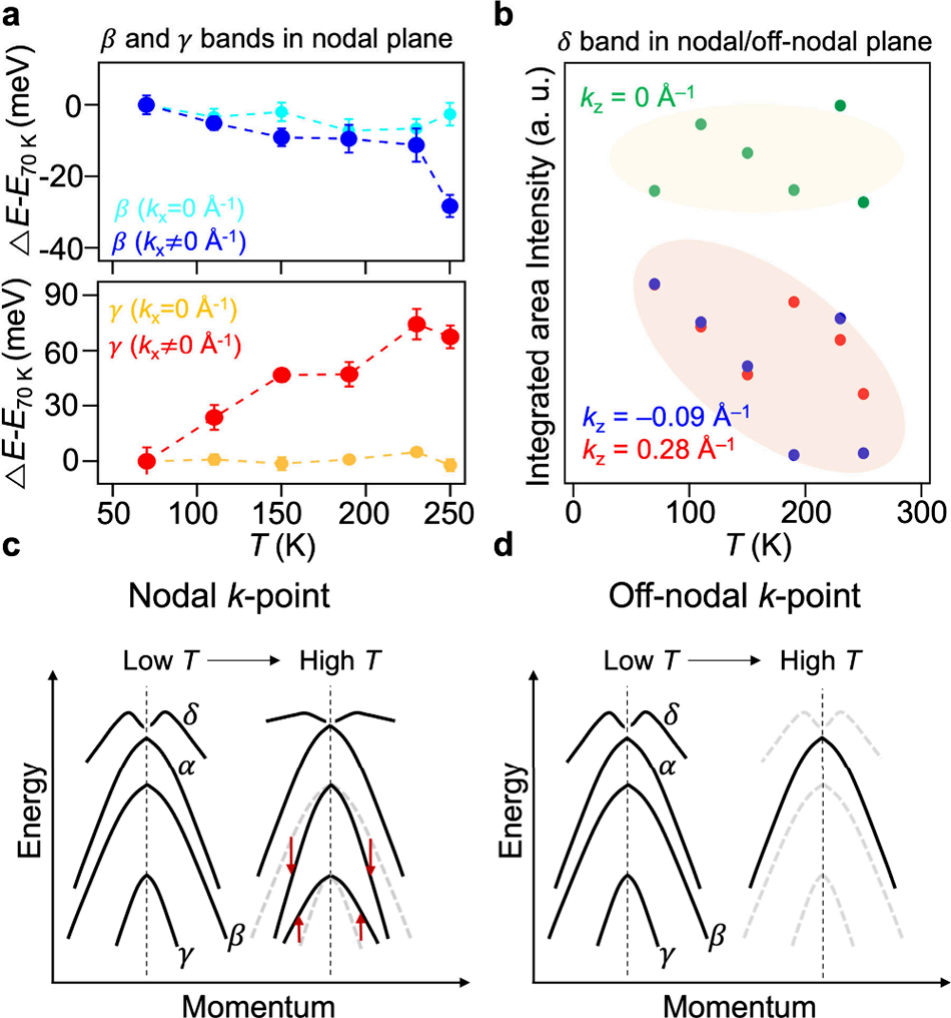
Temperature-dependent evolution in the peak positions and the ARPES spectral intensity. (a) Temperature-dependent energy shift of the β and γ bands, derived from the EDCs at *k*_*z*_ = 0 Å^–1^ taken at two different *k*_*x*_ values 0 and 0.16 Å^–1^ (see Figure S7 in SI). (b) Integrated area intensity at *k*_*z*_ = 0, −0.09, 0.28 Å^–1^ obtained from integrated EDCs over the momentum ranging from *k*_*x*_ = −0.2 to 0.2 Å^–1^ and the energy ranging from *E* – *E*_F_ = −0.1 to 0.1 eV, as illustrated by the red dashed box in Figure 3b. (c, d) Schematic illustration of the two distinct temperature responses of bands in nodal and off-nodal *k*-points.

The suppression of the spectral weight near *E*_F_ can be better quantified by the integrated spectral weight within a confined energy and momentum space (Figure S7d). Before integration, we ensure that the normalization procedure is adequately applied to guarantee a reliable comparison of temperature-dependent spectral weight changes (Figure S10). We focus on the intensity ranging from *E* – *E*_F_ = −0.1 to 0.1 eV and *k*_*x*_ = −0.2 to 0.2 Å^–1^, as outlined by the red-dashed rectangle in Figure 3(b) and the yellow rectangle in Figure S7d. As shown in Figure 4b, we found distinct temperature-dependent behaviors at different *k*_*z*_ values. At the nodal plane (*k*_*z*_ = 0 Å^–1^), the δ band maintains nearly constant intensity even at elevated temperatures. In contrast, at the off-nodal plane (*k*_*z*_ = 0.28 and −0.09 Å^–1^), the intensity from the δ band is predominantly suppressed at high temperatures.

Our results reveal two distinct temperature-dependent behaviors in the nodal and off-nodal planes. At the nodal plane (*k*_*z*_ = 0 Å^–1^), the most outstanding features in the temperature-dependent electronic structure changes are temperature- and momentum-dependent energy shifts, as illustrated in Figure 4c. Theoretical studies suggest that SOC-induced band splitting occurs across the nodal plane except for the zone center (*k*_*x*_ = 0 Å^–1^) at which the band remains degenerate due to symmetry constraints.^12,14,18^ Consequently, the temperature dependence at the nodal plane predominantly manifests itself as momentum-dependent energy shifts with fixed points at the zone center. Our findings are consistent with spectroscopic features often associated with itinerant magnetism, and they suggest that the itinerant nature of weak altermagnetism is an important ingredient to consider to fully understand altermagnetic materials. Even though our measurements could not precisely capture the band splitting, the observed trends align with and support the itinerant character proposed in the previous experimental work.^14^ However, at off-nodal planes, contrary to previous studies on bulk or thick films that report the merge of split bands into degenerate states,^14,15^ as the altermagnetic phase transitions to the paramagnetic phase, standout features in our ARPES data are significant electronic decoherence near *E*_F_ and higher binding energies rather than shifts in the energy-momentum positions of the bands, as presented in Figure 4d. These results deviate from the expected itinerant altermagnetic picture for MnTe and suggest a more complex underlying mechanism.

The decoherence of electronic states near *E*_F_ is often associated with the Heisenberg-type local magnetism.^27–33^ For example, in a collinear antiferromagnet FeTe^32^ with a large magnetic moment (∼2.1 μ_B_), a similar suppression of ARPES intensity has been observed.^30,31^ The substantial temperature- and momentum-dependent suppression of spectral weight may suggest that it is necessary to consider the local moment and spin fluctuations^29,30^ for a better understanding of the altermagnetism in MnTe. Our results also emphasize that it is important to consider the material-specific properties for a comprehensive understanding of altermagnetism in real materials. MnTe is a semiconducting antiferromagnet with a large magnetic moment (∼4 μ_B_),^34–36^ and the antiferromagnetic ordering is attributed to localized Mn 3*d* orbitals with superexchange interactions mediated by Te 5*p* orbitals.^37^ Therefore, it may be natural to consider the role of local magnetic moments to understand the altermagnetism in MnTe properly. On the other hand, RuO_2_, another intensely studied candidate material for altermagnetism, is a prototypical itinerant antiferromagnet.^6,16,38,39^ It exhibits typical metallic antiferromagnetic characteristics, including a very small magnetic moment of ∼0.05 μ_B_.^38,39^ The antiferromagnetism in RuO_2_ is mediated by superexchange interactions between half-filled 4*d* orbitals.^38^

The experimental observation of the out-of-plane momentum-dependent temperature responses in the electronic structure of MnTe suggests that a simplistic picture based on the itinerant magnetic interaction may not suffice to fully comprehend the complexity of this materials system. To answer whether such dichotomous temperature response is universal in altermagnets, or nodal vs antinodal is the only parameter determining such distinct temperature dependence, requires further experimental and theoretical endeavors. In particular, temperature-dependent magnetization measurements combined with theoretical calculations will provide deeper insights into the interplay between itinerant and localized magnetism in MnTe.

The momentum-dependent electronic property could be explained in terms of the momentum-dependent interactions stemming from the distinct wave symmetries, such as *d*-wave and *g*-wave, influencing momentum-dependent spin polarization and electronic band topology.^3,4^ A possible explanation is that at *k*_*z*_ = 0, the dominant contribution to spin-splitting originates from SOC, leading to a band structure that follows a relatively simple *d*-wave-like symmetry. In contrast, at *k*_*z*_ ≠0, the altermagnetic spin-splitting becomes more prominent, which may be associated with a more complex *g*-wave symmetry. Additionally, hybridization between Mn *d*- and Te *p*-orbitals is expected to play a more significant role at *k*_*z*_ ≠ 0, further modifying the electronic structure.^21^ Such momentum-dependent band hybridization, combined with altermagnetic spin splitting, could provide a hint to understanding the observed *k*_*z*_-dependent band characteristics.^14,15^

In conclusion, we have systematically investigated the momentum- and temperature-dependent evolution of the electronic structures in altermagnetic MnTe epitaxially grown on Si(111). Our ARPES results reveal a highly photon energy-dependent band structure and two distinct types of temperature response at different out-of-plane momentum planes. We observed, with increasing temperature, significant electron decoherence near *E*_F_ at off-nodal *k*_*z*_ planes, while energy-momentum shifts of the bands dominate the temperature dependence at nodal *k*_*z*_ planes. By revealing intricate relations between the electronic structure and the unconventional magnetic properties of the MnTe thin film, our work suggests that it is necessary to go beyond the prevailing itinerant picture to fully comprehend the electronic and magnetic properties of MnTe.

## Methods

### Thin Film Growth

MnTe thin film samples were grown using MBE at Beamline 10.0.1, Advanced Light Source, Lawrence Berkeley National Laboratory. The base pressure of the MBE chamber was 2 × 10^–10^ Torr. *n*-Doped Si(111) substrates were used. A well-ordered 7 × 7 phase of Si(111) was obtained by 20 cycles of flash annealing at 1200 °C. High-purity Mn and Te sources were simultaneously evaporated onto the substrate. The substrate temperature was kept at 300 °C during the growth. The growth process was monitored using *in situ* reflective high-energy electron diffraction (RHEED), and the deposition rate is approximately 15 min per layer.

### ARPES Measurements

The epitaxially grown MnTe thin films were directly transferred to the ARPES analysis chamber, without exposure to air, for *in situ* measurements in a vacuum of 4 × 10^–11^ Torr. The ARPES data were taken with a Scienta R4000 electron analyzer at temperatures ranging from 20 to 250 K. The energy and angular resolution were set to be approximately 15–20 meV and 0.1 degrees, respectively.

## Supplementary Material



## Notes

Core level measurement of epitaxially grown MnTe; Experimental geometry-dependent electronic structure of MnTe; Polarization-dependent electronic structure of MnTe; *k*_*z*_ dispersion of MnTe; Temperature-dependent α band; Temperature-dependent electronic structure of MnTe; Detailed temperature-dependent results; Multi-peak fitting results; Normalization before integration (PDF)
