# Supplementary material for: Dichotomous Temperature Response in the Electronic Structure of Epitaxially Grown Altermagnet MnTe
Source: Nano Lett. 2025 May 19;25(22):8969–75. doi: 10.1021/acs.nanolett.5c01158 (PMC12142661; doi:10.1021/acs.nanolett.5c01158)
Supplement: Supplementary file 1 [file nl5c01158_si_002.pdf]

Supplementary Information for

Dichotomous temperature response in the electronic structure of epitaxially-grown altermagnet MnTe

Ji-Eun Lee[1,2], Yong Zhong[1,3,4], Qile Li[5], Mark T. Edmonds[5,6], Zhi-Xun Shen[3,4,7], Choongyu Hwang[8,9], Sung-Kwan Mo[1*]*

[1]*Advanced Light Source, Lawrence Berkeley National Laboratory, Berkeley, CA 94720, USA*

[2]*Max Planck POSTECH Center for Complex Phase Materials, Pohang University of Science and Technology, Pohang 37673, Korea*

[3]*Stanford Institute for Materials and Energy Sciences, SLAC National Accelerator Laboratory, Menlo Park, CA 94025, USA*

[4]*Department of Applied Physics, Stanford University, Stanford, CA 94305, USA*

[5]*School of Physics and Astronomy, Monash University, Clayton, Victoria 3168, Australia*

[6]*ANFF-VIC Technology Fellow, Melbourne Centre for Nanofabrication, Victorian Node of the Australian National Fabrication Facility, Clayton, Victoria 3168, Australia*

[7]*Department of Physics, Stanford University, Stanford, CA 94305, USA*

[8]*Department of Physics, Pusan National University, Busan 46241, Korea*

[9]*Quantum Matter Core-Facility, Pusan National University, Busan 46241, Korea*

S1. Core level measurement of epitaxially grown MnTe

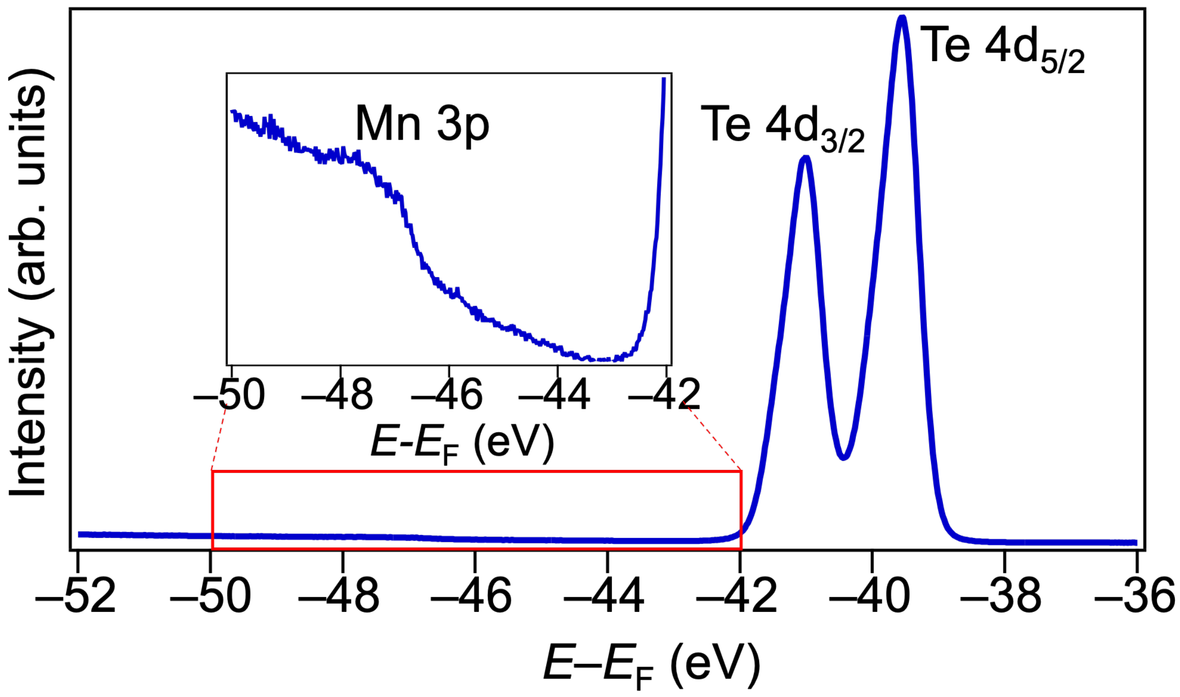

Figure S1. Core level photoemission spectrum of epitaxially grown MnTe, showing Te 4*d* and Mn 3*p* peaks. The red rectangular box highlights the magnified region for identifying the Mn 3*p* peak.

S2. Experimental geometry-dependent electronic structure

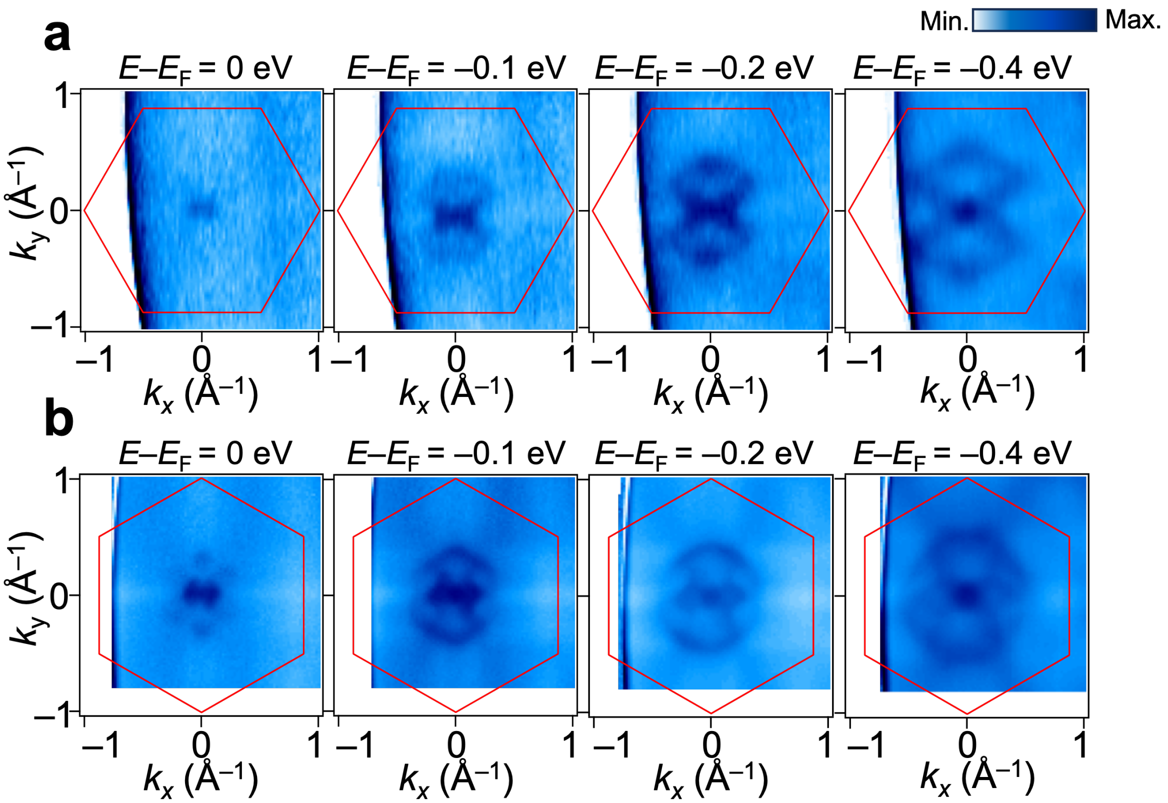

Figure S2. Constant energy maps measured at 55 eV corresponding to different *in-plane* angles in the ARPES experimental geometry setups. The *in-plane* angle of (a) is rotated by 90˚ relative to that of (b).

S3. Polarization-dependent electronic structure of MnTe

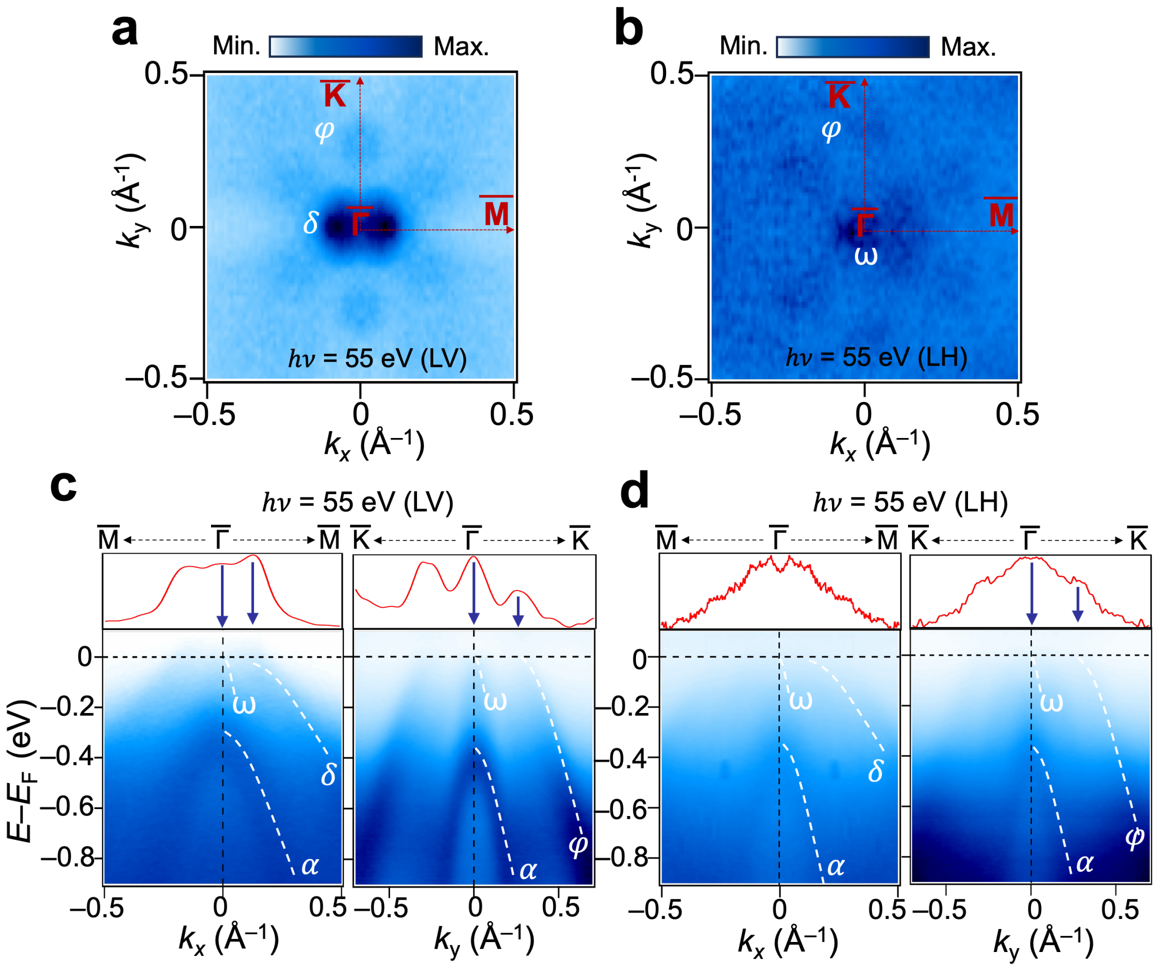

Figure S3. ARPES spectra taken at 55 eV with linear vertical (LV) and horizontal (LH) polarization. (a),(b) Fermi surfaces with LV and LH polarization. (c),(d) E-k dispersion along the $\overline{\Gamma}$-$\overline{M}$ and $\overline{\Gamma}$-$\overline{K}$ directions with LV and LH polarization.

S4. k_z dispersion of MnTe

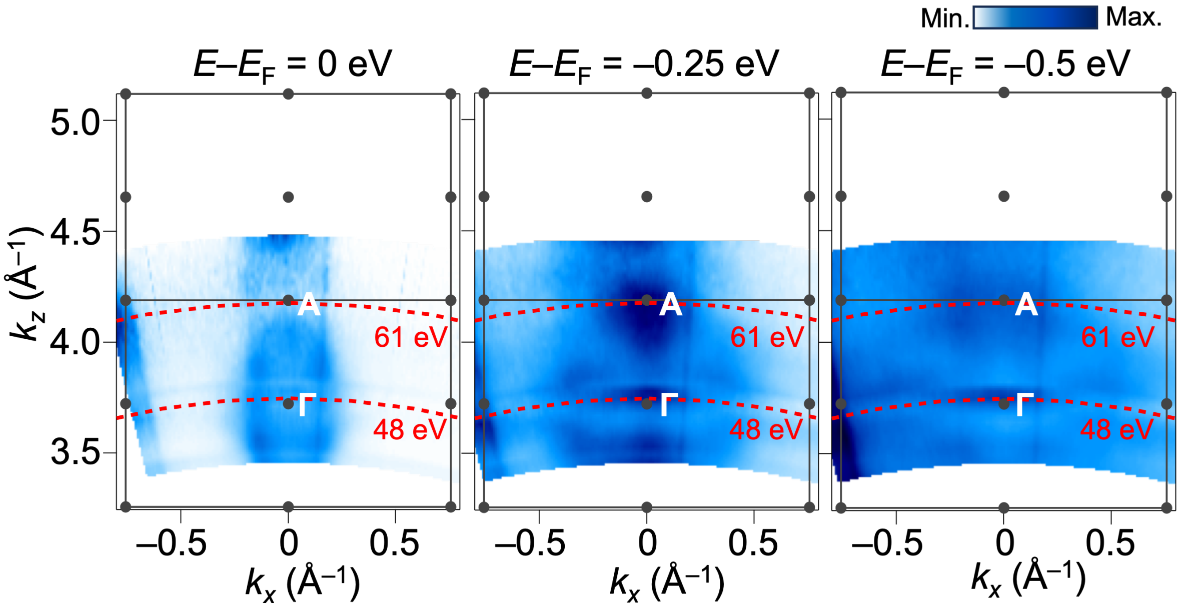

Figure S4. k_z dispersion of MnTe using photon energy ranging from 40 eV to 70 eV along the Γ-M direction. Constant energy maps of k_x-k_z plane at E–E_F = 0, –0.25, and –0.5 eV, respectively.

To determine the k_z momentum, we introduce a single potential barrier V_0, known as *inner potential*. The k_z momentum can be expressed as:

$$k_z = \sqrt{\frac{2m_e}{\hbar^2}(E_k cos^2\theta + V_0)}$$

where m_e is the effective mass of the electron in the sample, θ is the emission angle of the photoelectrons, and E_k is the kinetic energy of the photoelectrons. The kinetic energy E_k of the photoelectrons is given by:

$$E_k = hv - E_B - \emptyset$$

where hv is the photon energy used in the photoemission process, E_B is the electron binding energy, and $\emptyset$ is the work function.

By performing photon energy-dependent ARPES measurement, k_z dispersion can be resolved as shown in Figure S3. By investigating the periodicity of k_z at three binding energies (0, 0.25, 0.5 eV), we determine that inner potential V_0 is 10 eV.

S5. Temperature-dependent α band

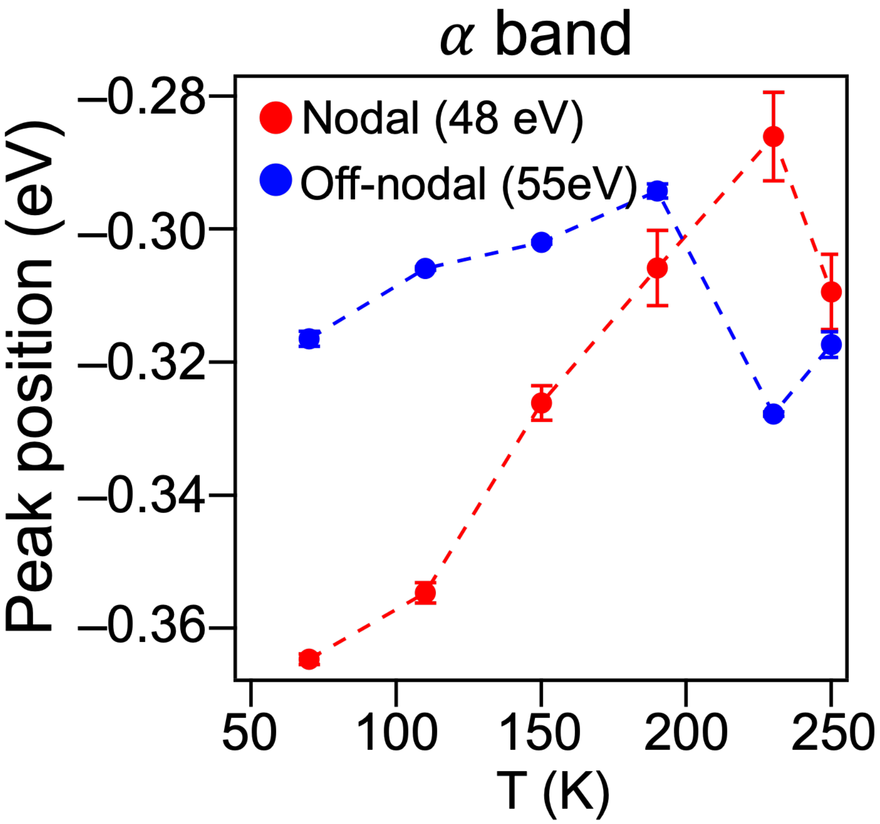

Figure S5. Temperature-dependent energy shift of the α band, obtained from EDCs extracted at k_x=0 Å^{-1} (Γ point), in the nodal and off-nodal planes. While the α band in the nodal plane shifts by ~55 meV from 70 K to 250 K, the shift in the off-nodal plane is negligible.

S6. Temperature-dependent electronic band structure of MnTe

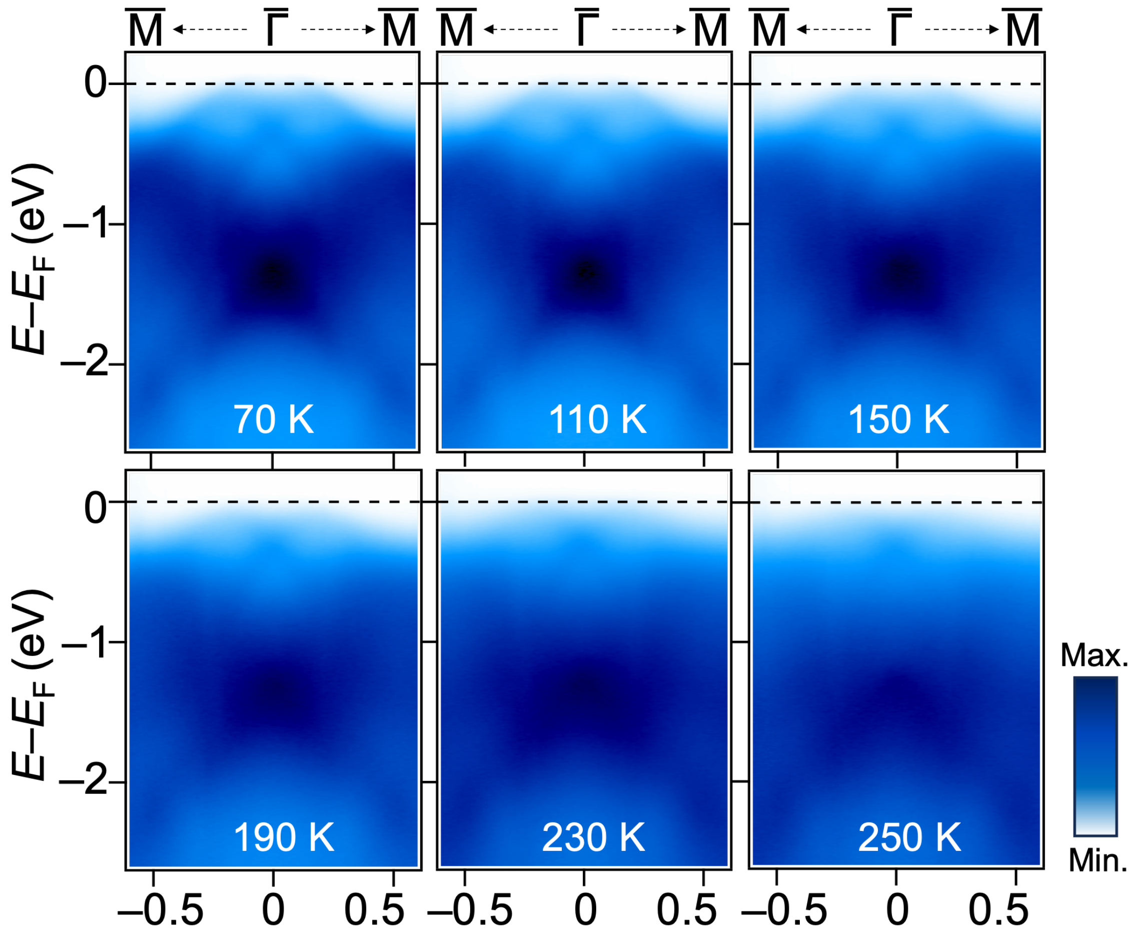

Figure S6. Temperature-dependent ARPES intensity cuts along the $\overline{\Gamma}$-$\overline{M}$ direction measured at 45 eV.

S7. Detailed temperature-dependent results

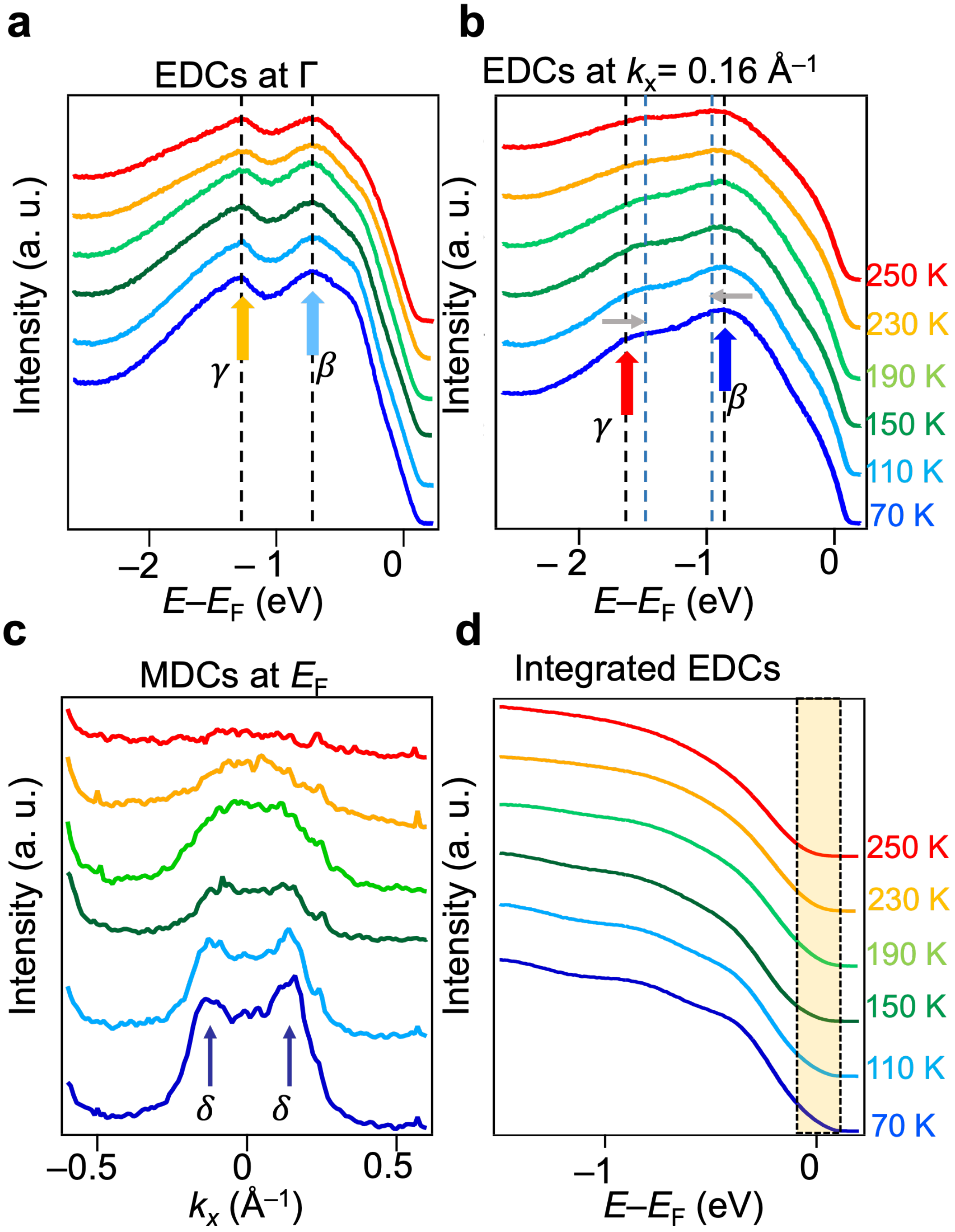

Figure S7. (a),(b) Temperature-dependent EDCs at $k_z = 0$ Å^{-1} taken at two different k_x values: (a) $k_x = 0$ Å^{-1} and (b) $k_x = 0.16$ Å^{-1}. (c) Temperature-dependent MDCs taken at E_F. Two double peaks

indicate the δ band. (d) Temperature-dependent integrated EDCs over the momentum ranging from $k_x = -0.2$ to 0.2 Å^{-1}, as indicated by the red-dashed rectangle in Figure 3b.

S8. Multi-peak fitting results

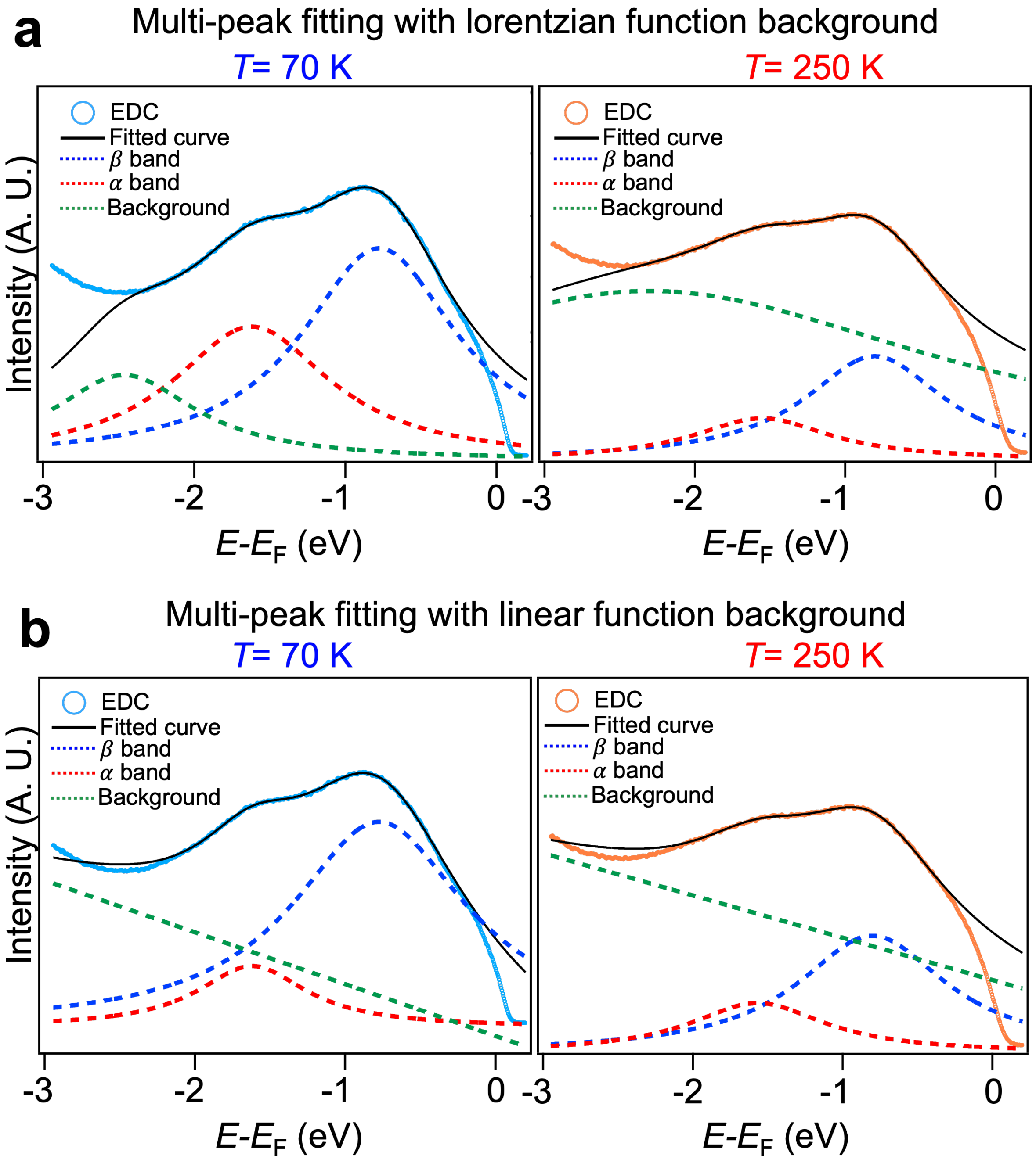

Figure S8. Multi-peak fitting results using (a) Lorentzian function and (b) linear function as background models at 70 K and 250 K. The consistency between the two methods confirms the reliability of the extracted energy shifts.

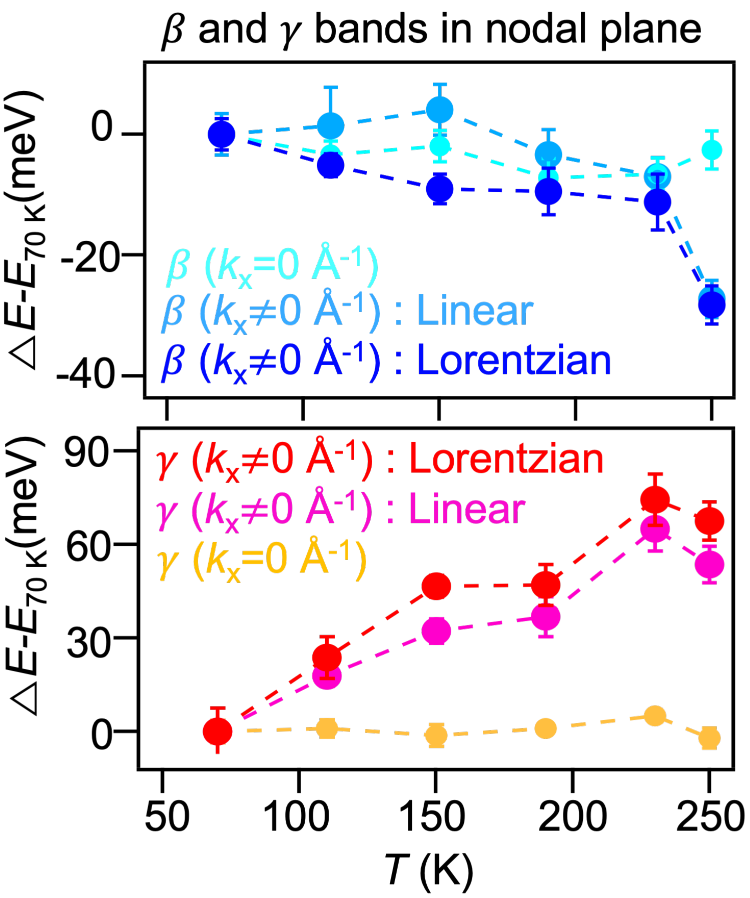

Figure S9. Temperature-dependent energy shifts of the γ and β bands. Despite minor numerical differences between the two fitting approaches, the overall trend remains consistent, confirming the reproducibility of our results.

S9. Normalization before integration

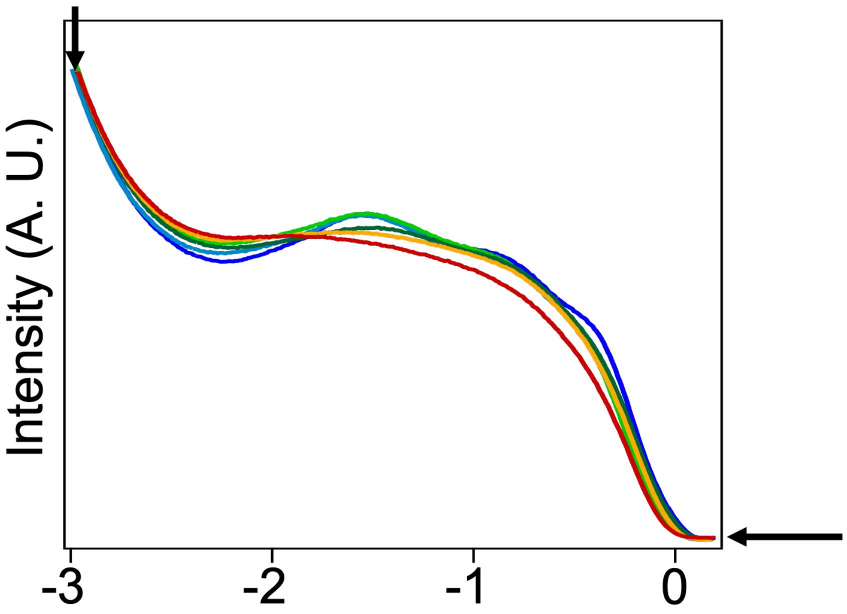

Figure S10. Normalization procedure, with arrows indicating two reference points used for scaling. The initial normalization at 0.1 eV provides a stable baseline, while the adjustment at -2.9 eV fine-tunes the intensity scaling.
